# A test of agent-based models as a tool for predicting patterns of pathogen transmission in complex landscapes

**DOI:** 10.1186/1472-6785-13-35

**Published:** 2013-09-25

**Authors:** Kelly E Lane-deGraaf, Ryan C Kennedy, SM Niaz Arifin, Gregory R Madey, Agustin Fuentes, Hope Hollocher

**Affiliations:** 1 Department of Biological Sciences, University of Notre Dame, Notre Dame, IN, USA; 2 Department of Computer Science & Engineering, University of Notre Dame, Notre Dame, IN, USA; 3 Department of Anthropology, University of Notre Dame, Notre Dame, IN, USA; 4 Department of Bioengineering and Therapeutic Services, University of California, San Francisco, USA; 5 Current address: Odum School of Ecology, University of Georgia, Athens, GA, USA

**Keywords:** Agent-based model, Dispersal, Pathogen transmission, Landscape heterogeneity, GIS

## Abstract

**Background:**

Landscape complexity can mitigate or facilitate host dispersal, influencing patterns of pathogen transmission. Spatial transmission of pathogens through landscapes, therefore, presents an important but not fully elucidated aspect of transmission dynamics. Using an agent-based model (LiNK) that incorporates GIS data, we examined the effects of landscape information on the spatial patterns of host movement and pathogen transmission in a system of long-tailed macaques and their gut parasites. We first examined the role of the landscape to identify any individual or additive effects on host movement. We then compared modeled dispersal distance to patterns of actual macaque gene flow to both confirm our model’s predictions and to understand the role of individual land uses on dispersal. Finally, we compared the rate and the spread of two gastrointestinal parasites, *Entamoeba histolytica* and *E. dispar*, to understand how landscape complexity influences spatial patterns of pathogen transmission.

**Results:**

LiNK captured emergent properties of the landscape, finding that interaction effects between landscape layers could mitigate the rate of infection in a non-additive way. We also found that the inclusion of landscape information facilitated an accurate prediction of macaque dispersal patterns across a complex landscape, as confirmed by Mantel tests comparing genetic and simulated dispersed distances. Finally, we demonstrated that landscape heterogeneity proved a significant barrier for a highly virulent pathogen, limiting the dispersal ability of hosts and thus its own transmission into distant populations.

**Conclusions:**

Landscape complexity plays a significant role in determining the path of host dispersal and patterns of pathogen transmission. Incorporating landscape heterogeneity and host behavior into disease management decisions can be important in targeting response efforts, identifying cryptic transmission opportunities, and reducing or understanding potential for unintended ecological and evolutionary consequences. The inclusion of these data into models of pathogen transmission patterns improves our understanding of these dynamics, ultimately proving beneficial for sound public health policy.

## Background

An ongoing need exists for an enhanced toolkit for predicting spatial patterns of pathogen transmission [1-4]. While current models incorporate spatial aspects of the host [5,6], pathogen [7-9], or more rarely, both [10,11], many current models of infectious disease ignore the more complex landscape features, including interactions between hosts, which can be mitigated or facilitated by landscape complexity [12,13]. Pathogen transmission potential is an integrated measure of both infectivity and an individual’s opportunity for encountering the pathogen in the environment or through contact with other infectious individuals [14]. Therefore, models of pathogen infection must examine this transmission potential and focus on how landscape features directly influence this potential and the resulting patterns of pathogen spread. The continued shift in research emphasis towards efforts concentrating on the underlying ecological determinants and spatial dynamics of pathogen transmission will result in more effective global public health policy [15-17].

Employing geographic information systems (GIS) data as a tool in epidemiologic analyses is not new, given the ability of GIS to incorporate spatial and non-spatial data in one system [18]. Colwell and colleagues (1996) successfully implemented research programs using GIS data of Bangladesh to more completely understand the transmission of *Vibrio cholerae* by modeling it as a component of the environment. Outbreaks were shown to be both seasonal and geographically localized, influenced strongly by the presence of estuaries and major rivers [19]. Modeling of pathogen transmission and spread of infectious diseases with a focus on GIS analysis has been undertaken in several outbreaks and epidemics, including plague (*Yersinia pestis*) in the Southwestern United States, rabies in Trinidad, and Chagas disease vectors in Colombian villages [20-22]. These studies demonstrate that analysis of pathogen transmission patterns is enhanced through the flexibility in analyzing spatial data inherent to the GIS system.

Recently, agent-based models (ABMs), or individual-based models, have been effectively employed as an enhanced tool to address the spatial dynamics of pathogen transmission [7,23,24]. These models explicitly represent individual entities in the system under study and can realistically accommodate extreme heterogeneity among the agents by allowing individuals to incorporate spatial interactions into the simulations directly [25]. This flexibility permits ABMs to account for population outliers and long-tailed distributions and to model rare, albeit important, events in the system under study [7]. Agent-based modeling is therefore ideal for addressing complex questions regarding how hosts and pathogens navigate a complex landscape. Recently developed ABMs have been used to elucidate infectious disease dynamics in systems as disparate as demonstrating the process of granuloma formation following a tuberculosis infection [26], evaluating influenza vaccination strategies in Italy, with a focus on implementation campaigns mitigating a global pandemic to H5N1 [8], and understanding the relationship between vector ecology, human behavior, and spread of African sleeping sickness [10].

### Host behavior and ecology

Macaque species are found throughout Asia and in parts of Africa, with the *fascicularis* subgroup having an extensive range throughout much of Southeast Asia. Long-tailed macaques (*Macaca fascicularis*) thrive in a variety of habitat types, including forests, grasslands, semi-deserts, and most especially, urban landscapes [27], often living commensally with humans. While macaques are generally considered to be frugivorous, long-tailed macaques are known to have a highly flexible diet and can be considered, in parts of their range, to be omnivorous. Male dispersal is common, while females remain in their natal group. Little is known about dispersal duration or distance [27]; however, long-distance dispersals have been documented [28]. Gene flow between population groups is maintained by male dispersal as well as by group fission events, especially common as population size increases. Thus, long-tailed macaques thrive in complex, anthropogenic landscapes and can disperse across wildly variable habitats.

On the island of Bali, Indonesia, a system of temple complexes act as core use areas for long-tailed macaques (*Macaca fascicularis*) [29-32]. While the macaques’ home ranges extend well beyond the confines of the temple complexes, a substantial segment of a given population can be found in and around these temples on a regular basis. Dispersing male macaques may act as both units of gene flow between seemingly isolated macaque populations and as mechanisms of pathogen transmission across the island [29]. Human land use patterns have resulted in a mosaic of riparian forest, small forest patches, agricultural lands, and urban areas across much of the island. The broad distribution of macaque populations on Bali suggests that macaques use this human-modified landscape by exploiting agriculturally-dominated, riverine links between populations for dispersal and the sanctuary nature of temples as stabilized food resources [29]. This protection and resource availability has allowed macaques to exist in moderately high densities alongside high human densities [31].

### Pathogen ecology and epidemiology

Gastrointestinal parasites are among the most prevalent suite of parasites and pathogens globally, with representatives found in nearly all mammal species and causing morbidity in nearly all individuals at some point in their lifetime [33]. The success of this suite of parasites is due, in large part, to their mode of transmission. Relying on the fecal-oral route and often occurring with environmentally stable infective stages, infectious agents pass through the gut of an infected individual, are deposited in water or on plant matter, and are ultimately consumed, completing the transmission cycle [34]. The environmentally stable infective stage makes the spatial transmission of gastrointestinal parasites of special relevance. Landscape type and quality have been shown to be important in the prevalence and intensity of intestinal parasites [35-37]. For example, intestinal parasite burden was significantly greater in low quality, fragmented habitat in populations of two species of howler monkeys (*Alouatta palliata* and *A. pigra*) [38].

It is estimated that more than 500 million people are infected with at least one species of *Entamoeba* at any given moment [34]. Infection rates increase with impoverished economies and lack of access to clean drinking water. Both *Entamoeba histolytica* and *E. dispar*, along with at least two other amoebas (*Iodamoeba* and *Endolimax*) infect humans, domestic animals, and wildlife species, including non-human primates [34,35,38,39]. While *E. histolytica* is linked to numerous cases of diarrhea and more than 100,000 human deaths/year, *E. dispar* is largely un-symptomatic, causing neither disease nor tissue degeneration [40]. Both species of *Entamoeba* have been found in macaques throughout their range, including on the island of Bali, Indonesia [35,41,42]. The similarity in transmission strategy and phylogeny coupled with highly disparate disease severities makes *E. histolytica* and *E. dispar* an ideal model system for examining the effect of landscape variability on host dispersal and pathogen transmission.

### Modeling host movement and pathogen transmission

LiNK, the ABM presented here, incorporates landscape features critical to understanding pathogen transmission patterns by using GIS layers of the actual system’s landscape [29,43]. The powerful spatial analysis permitted through the use of GIS data combined with the strength and utility of ABM provides a mechanism to understand the spatial context critical for understanding patterns of pathogen transmission. LiNK has the ability to generate predictions regarding host dispersal and pathogen distributions based on the anthropogenic landscape, human-wildlife interactions, host behaviors and interactions, and pathogen life histories at island-, population-, and individual levels.

Here, we present an agent-based model of host (macaques) and pathogen (gastrointestinal parasites) movement through the Bali landscape. First, we aim to determine the impact of the inclusion of landscape information on patterns of macaque dispersal. We hypothesize that the inclusion of landscape information into our model will alter the dispersal pattern of macaques from isolation by distance, as predicted in the absence of landscape information, to one of dispersal linked by habitat type. We then compare the difference between modeled dispersal patterns generated with the inclusion of landscape information to that of actual macaque gene flow patterns, as measured by genetic distance. We hypothesize that the inclusion of landscape information into our model of macaque dispersal will correlate better to measured genetic distance than when landscape information is excluded. Finally, we explore the likely path and rate of pathogen transmission of two gastrointestinal parasites – *Entamoeba histolytica* and *E. dispar* – modeled using varying pathogen virulence, infectivity, and infectiousness parameters. We hypothesize that the inclusion of landscape information will result in environmental context dependence in rate and route of infection, with landscape heterogeneity mitigating overall infection. We also hypothesize differences between the two parasites independent of landscape features with the less virulent parasite – *E. dispar* – reaching overall greater distances from the site of initial infection due the host’s ability to maintain dispersal patterns as though healthy.

## Methods

### Model description

**Figure 1:**
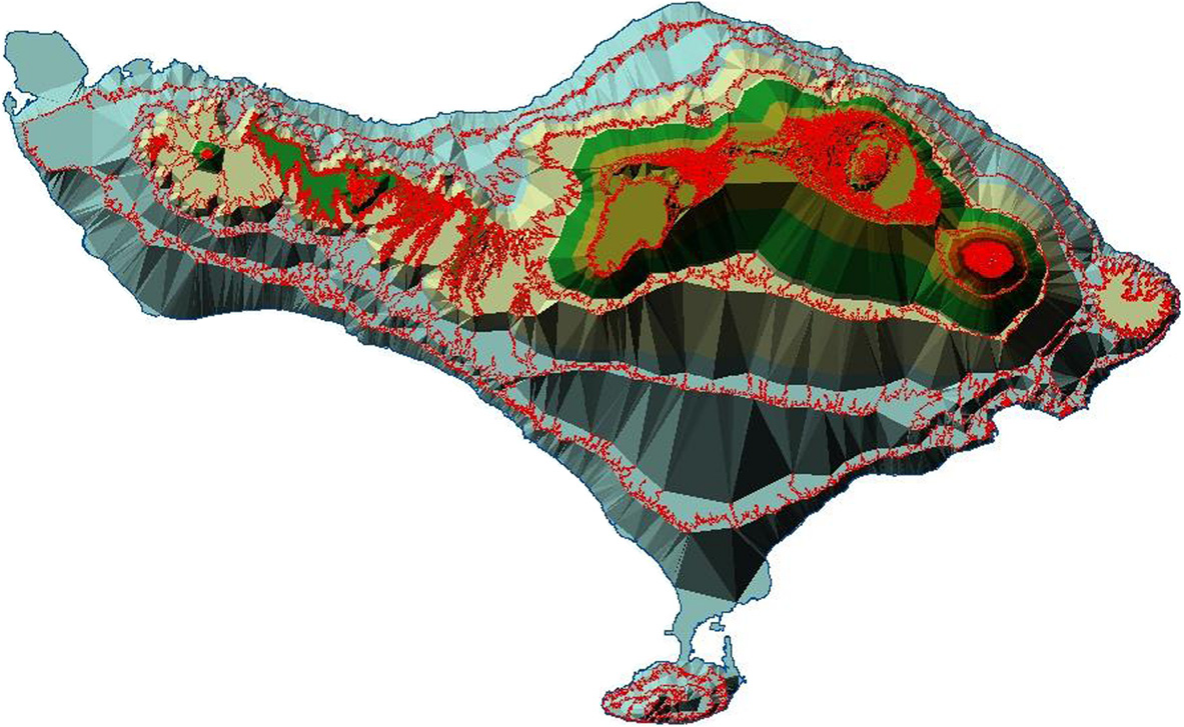
**Map of Bali, based on GIS data (Southern et al. **[32]**), with forest-covered areas shown in green/green-drab and elevation gradients shown in relief.** Red lines indicate areas of sharp changes in slope.

LiNK is an agent-based model created and implemented using GIS maps of Bali [42]. GIS layers include data on coastline, rivers and lakes, forest, rice agriculture, urban areas, roadways, and temple locations (see Figure 1), created via remote sensing and GPS ground-checking. Within LiNK, macaques, or agents, 'live’ in the landscape making reproductive and dispersal decisions based on behavioral data known at the model’s development [27,30,31]. Decisions to disperse are based on male macaque age, with dispersal occurring between the ages of six and eight. Once a macaque has left his natal population, all decisions regarding direction and feasibility of travel are based on both prior location and the landscape parameters provided by the GIS layers. Agents travel in a direction that maximizes their time spent in preferred landscape types while avoiding the last occupied location.

**Figure 2:**
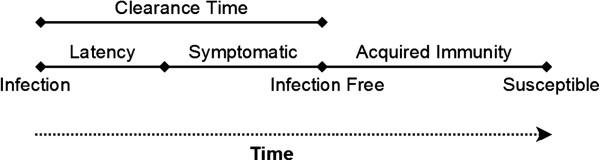
**Timeline of infection in model showing the relationship between pathogen parameters in our simulation.** Depending on the parameters used, macaques can become permanently immune to a pathogen.

Transmission dynamics are modeled based on macaque dispersal and a set of variable parameters specific to the pathogen of interest: virulence, infectivity, infectiousness, latency period, clearance time, and immunity development. Latency period, clearance time, and immunity development are timing parameters, described in Figure 2. Virulence is modeled as the pathology of the parasite, or the level of agent or macaque illness required to optimize transmission of the parasite. This parameter impacts both movement ability and survivorship. Infectivity is modeled as the area around an infected agent in which another agent is at risk of becoming infected. While a measure of distance, infectivity remains independent of a unity of distance due to the conversion necessary for applying the distance across latitude and longitude. Infectiousness is modeled as the likelihood of infecting a susceptible agent.

### Model development details

LiNK is a spatially explicit model that consists of agents representing macaques and GIS layers representing the landscape including the coastline of Bali, cities, forests, rivers and lakes, rice fields, roads, macaque temple sites, and 100 ha buffer zones around macaque temples. At each time step, agents evaluate potential new positions, noting their current landscape and directional bias. Dispersing macaques enter temples, depending on their proximity to individual temples in the landscape. Female macaques have a 25% chance to give birth annually from 3–13 years of age. Macaque movement through the landscape was implemented based on previous findings [29,31], including dispersal distances and habitat preferences. Male dispersal and female philopatry were confirmed through sequencing of mitochondrial and *Y* DNA loci [29]. This movement through weighted probabilities facilitates the overall purpose of the model – to understand how landscape dynamics influence host movement.

Patterns of infection spread throughout Bali emerge over time, in accordance to host movement through the landscape. Macaques are aware of their current and surrounding landscape, which they use to make movement decisions. Macaques interact with other macaques both while dispersing and within temples. Upon infection, the pathogen is in a latent period, which refers to the length of time before the infection becomes symptomatic. After completing the symptomatic phase, a macaque will become infection free and clear the pathogen, which prevents further transmission of the pathogen. Immunity development occurs after the infection has been cleared from individual macaques, ending when the again becomes susceptible. However, if this parameter is set to zero at initialization, re-infection can occur immediately after clearance. Transmission of the pathogen between macaques depends largely on infectiousness and infectivity. Infectivity is described as the transmission ring which both macaques have to be within to transfer infection; infectiousness is the likelihood that an infection will occur. Virulence is a measure of the severity of the infection. Higher virulence infections lower the chance of movement of the infected macaque.

The model was coded in Java [44] with the Repast simulation toolkit [45]. Repast and OpenMap [46] were utilized to display the model, while GeoTools [47] and JTS Topology Suite [48] were used to interact with the spatial information. The choice of tools used in this study was primarily driven by the necessity to process and visualize GIS data and to be cross-platform and open-source, where possible. For a more technical discussion of the algorithms used in defining these measures and for a more detailed description on model building, see the Overview, Design Concepts, and Details (ODD) available as an Additional file 1, which follows the protocol for describing agent-based models as suggested by Grimm et al. [43,49].

### Simulation experiments

#### Model verification & validation

Two main categories of model verification and validation were performed – internal sensitivity analyses and external confirmation of model predictions. Internal sensitivity analyses were further partitioned into two components – system functionality and biological relevance. System functionality analyses have been previously described [50] and will not be discussed here. Biological relevance analyses determined the effects of incorporating landscape data into the transmission model. All scenarios were repeated at four specific initial infection sites: two temples with population sizes greater than 300 individuals (Padangtegal or PU and Alas Kedaton or AK) and two temples with population sizes less than 60 individuals (Alas Nengahn or AN and Mekori or MK). These sites can also be partitioned by relative landscape heterogeneity, with two sites in largely homogeneous landscapes (Alas Kedaton or AK and Alas Nengahn or AN) and two sites in predominantly heterogeneous landscapes (Padangtegal or PU and Mekori or MK). We report the overall number of infections, the number of infections occurring in males and females, and the number of deaths occurring due to age, dispersal risk, and infection.

Two major sets of biological relevance analyses were performed: baseline landscape inclusion tests and pathogen parameterization tests. In the baseline landscape inclusion tests, we ran 50 replicates at each site of initial infection and compared the above three metrics occurring first with all GIS layers included and then with only the coastline available (no landscape data included). Using R, t-tests were performed to compare results by population size and by landscape heterogeneity. An ANOVA was used to compare individual death types as a function of inclusion of landscape data. The effects of individual landscape layers (forest, rice agriculture, urban areas, and buffer zones) were also compared across population size and landscape heterogeneity in order to assess the impact of specific landscape layers on macaque dispersal and pathogen transmission in our model. In the pathogen parameterization tests, we compared the above three metrics across high (85 out of 100), moderate (50 out of 100), and low (15 out of 100) levels of virulence, infectivity, and infectiousness, with 200 replicates of the model run for each scenario. The results of both the baseline landscape inclusion and pathogen parameterization verification and validation analyses are presented in an Additional file 2.

### External verification & validation

#### Landscape effects

To determine the overall role of the landscape on macaque movement, we compared the results of a series of simulations with all landscape information available, only landscape information about the coastline available (required for all simulations), and with individual landscape components removed, e.g. with the GIS layer representing forest cover removed. In this analysis, the total number of infections were recorded, with the results from 200 replicates/initial starting population averaged and compared with t-tests, for the analysis of all landscape information (ALL) versus no landscape information included (COAST ONLY), and ANOVA, for the analysis of effects of removing individual landscape information layers. Differences in infection pattern were also compared using a Mantel test, with an isolation-by-distance model hypothesized if the inclusion of landscape data plays no role in macaque movement [51,52].

#### Macaque dispersal

Given that macaque dispersal distance is expected to correlate with genetic distance [53-55], our next analysis compared genetic distance to modeled dispersal distance to externally confirm our predictions of landscape influenced macaque dispersal patterns. To do this, we parameterized the model to record dispersal events initiated at five populations located across the island. Dispersal events were recorded as the number of successful entries into each new population from the origination site, averaged over 300 replicates. We repeated the analysis including and excluding landscape information. Using R, we performed a Mantel test to determine statistical similarity between our modeled dispersal distance and measured genetic distance from genetic analysis of 15 macaque populations throughout Bali, using microsatellite analysis across 13 loci [29], Lane-deGraaf et al., unpublished data. All collections were approved by the University of Notre Dame IACUC (protocol 07–001 and 09–011) and the Indonesian Institute of Science (permit number 662.02/1090.DIII).

#### Pathogen transmission

Two gastrointestinal parasites were used to examine the direct impact of the inclusion of landscape information on parasite transmission. In this analysis, pathogen parameter values were set to represent *E. histolytica* and *E. dispar*[56], with the major differences focused on virulence, infectivity, and infectiousness (See Table 1). Two hundred replicates of each infection scenario were performed. For each parasite, we used t-tests to compare the number of overall infections occurring while varying the initial infection sites across landscape heterogeneity and population size (see above population descriptions). We also measure the number of infections occurring in each major landscape type (forest, rice agriculture, and urban areas), using t-tests to compare *E. histolytica* and *E. dispar* spread. Finally, we use ANOVA to compare the distance traveled from initial infection site, determining spread rate by averaging the number of infections reaching each temple.

**Table 1:** **Values used to parameterize model for the analysis of *E. histolytica *and *E. dispar *spread across Bali**

**Parameter**	**Value**	**E. histolytica**	**E. dispar**
Virulence	(0–100 range)	75	20
Infectivity	(0–100 range)	60	35
Infectiousness	(0–100 range)	60	40
Latency Period	Variable, in timesteps	7 timesteps	7 timesteps
Clearance time	Variable, in timesteps	28 timesteps	28 timesteps
Immunity Time	Variable, in timesteps	120 timesteps	120 timesteps
Natural resistance	(0–100 range)	1	1

## Results

### Landscape effects

**Figure 3:**
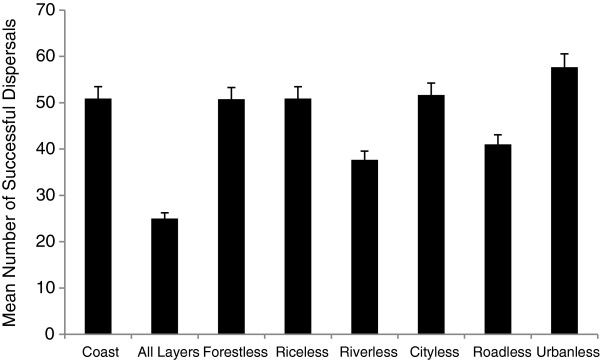
**Average number of infections/temple site with the exclusion of specific landscape layers.** Exclusion analysis began with only the coastline GIS layer, with all layers included, and finally with each layer cycled off independently. Note the substantial disparity between replicates with only the coast available and with all layers available as well as the increase in infection when the urban layers (road and city combined) were left off.

Significant differences were found in the number of temple sites reached by infection between analyses that included and excluded landscape information (t = -3.037, p < 0.01), with a greater number of temples reached in the absence of landscape data (Figure 3). This suggests that macaques using landscape information are limited in their movement by landscape barriers. When examining the effects of individual landscape layers, we found that the exclusion of data on rivers led to a significant reduction in temples reached via dispersal (F = 2.769, p < 0.012), while the exclusion of urban landscape information (which includes the combined effects of roads and cities) resulted in a significant increase in dispersal distances (F = 2.332, p < 0.014), reaching a greater number of temples and at a greater distance from their site of origin. This suggests that inclusion of additional landscape information identifies an emergent property of the landscape itself, with the combined urban landscape responsible for an increase in dispersal distances greater than would be predicted based on the roads or cities layers independently (Figure 3). Finally, the inclusion of all available landscape information resulted in a significant reduction in overall dispersal distances and number of temples reached (F = 2.462, p < 0.02).

### Macaque dispersal

Significant correlations were found between genetic distance and modeled dispersal distance from each of the five sites of origination when landscape information was included in the analysis, located in the north (correlation value = 0.33, p = 0.034), south (correlation value = 0.49, p = 0.008), east (correlation value = 0.40, p = 0.014), west (correlation value = 0.34, p = 0.028), and center (correlation value = 0.52, p = 0.002) of the island. However, in the absence of landscape information, no correlations were observed between genetic distance and modeled dispersal distance at any of the five sites of origination (N: correlation value = 0.01, p = 0.394; S: correlation value = 0.01, p = 0.411; E: correlation value = 0.01, p = 0.342; W: correlation value = 0.003, p = 0.522; C: correlation value = 0.01, p = 0.466). The tightest relationship between measured genetic distance and modeled dispersal distances occurred at the center, most anthropogenically-complex landscape on the island – Padangtegal (PU) – suggesting that macaques in LiNK are making landscape-driven decisions in much the same way as the long-tailed macaques of Bali.

### Pathogen transmission

**Figure 4:**
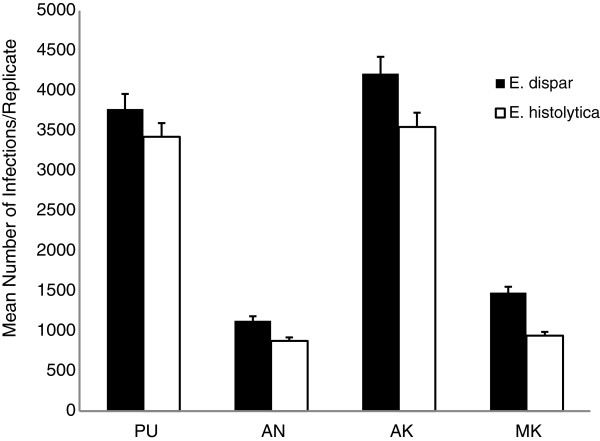
**Differences in modeled infection rates of *E. histolytica *and *E. dispar*, originating from four sites in Bali, Indonesia.** Each analysis is the comparison of the number of infections occurring when modeling *E. histolytica* or *E. dispar*, by initial site of infection (PU: t = 27.0996, p <2.2e^-16^; AN: t = 2.5733, p = 0.01164; AK: t = 22.505, p <2.2e^-16^; MK: t = 2.7825, p = 0.006519). Standard error bars shown; d.f. = 199 for each site of analysis.

**Figure 5:**
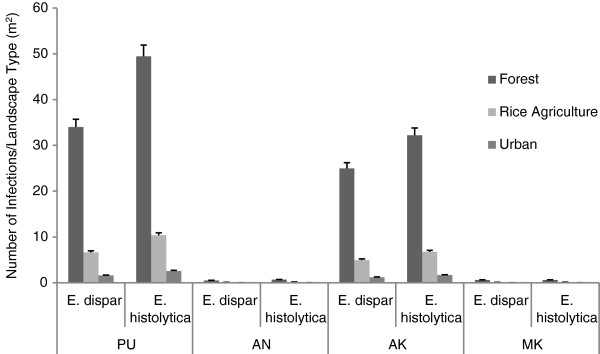
**The mean number of infections occurring island-wide, originating at one of 4 sites, reported as the number of infections occurring in each dominant landscape type.** Values are reported as the number of infections per m^2^ of habitat type surrounding the infection site. Significant differences occur between *E. histolytica* and *E. dispar* in all landscape types when infection originated at PU (See Table 2 for t and p values). Significant differences in rate of infection between parasites also occurred when infection originated at AK, but only in urban areas. Standard error bars shown.

The number of overall infections was greatest when infection originated in either Padangtegal (PU) or Alas Kedaton (AK) – the two largest macaque populations. Landscape heterogeneity had no overall impact at this level of analysis. At all initial infection sites, *E. dispar* infections significantly outnumbered *E. histolytica* infections (PU: t = 27.09, p <2.2e^-16^; AN: t = 2.5733, p = 0.01; AK: t = 22.51, p <2.2e^-16^; MK: t = 2.78, p = 0.006; Figure 4). When infection was examined by GIS layer, we found that infections were the most numerous in the forest dominated landscape. Rice agriculture lands and urban areas had infection occurring at lower levels (Figure 5). *E. histolytica* infections occurred at a significantly higher rate than *E. dispar* infections, in all landscape types and at each initial site of infection except Mekori (MK) where there was no difference in infection rate (Figure 5, Table 2).

**Table 2:** **T-tests and p values associated with Figure**5**, comparing rate of infection occurring in dominant landscape types when infection originated at each of 4 sites of initial infection**

**Comparison**	**t value**	**p value**
***PU:***		
Forest	**2.2824**	**0.03562**
Rice Agriculture	**2.9737**	**0.006431**
Urban Area	**6.3517**	**4.038e**^**-8**^
***AN:***		
Forest	1.4487	0.1508
Rice Agriculture	1.8208	0.07183
Urban Area	1.3017	0.1962
***AK:***		
Forest	1.0147	0.3320
Rice Agriculture	1.7067	0.1085
Urban Area	**6.0492**	**3.682e**^**-7**^
***MK:***		
Forest	1.5944	0.1144
Rice Agriculture	1.2429	0.2170
Urban Area	1.1987	0.2337

For each analysis, df = 199. Significant differences are bolded.

**Figure 6:**
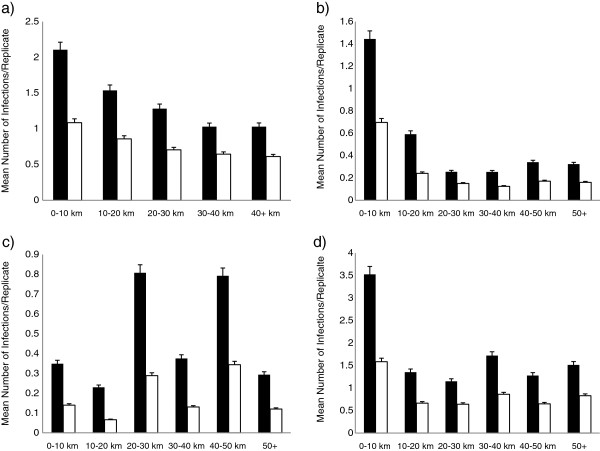
**Infection rates of *E. histolytica *and *E. dispar *partitioned by distance from site of initial infection: a) PU, b) AN, c) MK, and d) AK.** (For F and p values from ANOVA, see Table 3.) Peaks in infections in both parasites occur at a distance not immediately surrounding the initial infection site in at least two of the four populations – MK and AK. Dark bars are *E. dispar* infections; light bars rare *E. histolytica* infections.

At all sites, *E. dispar* reached greater distances at a significantly higher rate than *E. histolytica* (Figure 6, Table 3), supporting our hypothesis of more virulent pathogens limiting dispersal. However, distances with the highest rate of infection were not 0–10 km from the initial infection site, as predicted. At Mekori (MK), significantly greater numbers of infections reached temples at distances of 20–30 km and 40–50 km than any other distance, including 0–10 km from the initial site of infection. At Alas Kedaton (AK) and Alas Nengahn (AN), increases in the number of infections occurred in more distant temples. AK had infection peaks at temples 30–40 km and temples greater than 50 km from the initial infection site, and AN had infection peaks at temples greater than 40 km from the initial site of infection. These increases in infections at distances not immediately surrounding the site of initial infection suggests that landscape heterogeneity plays an important role in shaping the movement of macaques and pathogens through the landscape.

**Table 3:** **ANOVA results comparing *E. dispar *and *E. histolytica *spread from four sites of initial infection (Figure**6**)**

**Comparison:**	**F value**	**p value**	**Tukey HSD & Distance Category**
***E. dispar *from PU**	85.533	<2.2e^-16^	0-10 km* from 10–20 km, 30–40 km;
			10-20 km* from 20–30 km, 40–50 km;
			20-30 km* from 30–40 km;
			30-40 km* from 40–50 km
***E. histolytica *from PU**	49.84	<2.2e^-16^	0-10 km* from 10–20 km, 30–40 km;
			10-20 km* from 20–30 km, 30–40 km, 40–50 km;
			20-30 km* from 30–40 km, 40–50 km;
			30-40 km* from 40–50 km
***E. dispar *from AN**	27.74	<2.2e^-16^	0-10 km* from all other distances;
			10-20 km* from all other distances
***E. histolytica *from AN**	16.311	5.773e^-16^	0-10 km* from all other distances;
			10-20 km* from 20–30 km, 30–40 km
***E. dispar *from AK**	57.551	<2.2e^-16^	0-10 km* from all other distances;
			10-20 km* from 30–40 km;
			20-30 km* from 30–40 km, 50+;
			30-40 km* from 40–50 km
***E. histolytica *from AK**	30.841	<2.2e^-16^	0-10 km*from all other distances;
			10-20 km* from 30–40 km;
			20-30 km* from 30–40 km, 50+ km;
			30-40 km* from 40–50 km
***E. dispar *from MK**	26.886	<2.2e^-16^	0-10 km* from 20–30 km, 40–50 km;
			10-20 km* from 20–30 km, 40–50 km;
			20-30 km* from 30–40 km, 50+ km;
			30-40 km* from 40–50 km;
			40-50 km* from 50+ km
***E. histolytica *from MK**	30.841	<2.2e^-16^	0-10 km* from 20–30 km, 40–50 km;
			10-20 km* from 20–30 km, 40–50 km;
			20-30 km* from 30–40 km, 50+ km;
			30-40 km* from 40–50 km;
			40-50 km* from 50+ km

Asterisks denote significant differences between distance categories.

## Discussion

The incorporation of host movement and dynamic pathogen parameters combined with the inclusion of GIS data into our ABM allowed us to generate predictions of how macaque dispersal would be influenced by the landscape. In comparing these predictions against genetic evidence, our model was fully supported in predicting patterns of gene flow across the landscape. Our results show that an ABM which incorporates landscape information generates patterns of dispersal that accurately reflect actual dispersal patterns, as measured by genetic distance between populations. Most importantly for this system, and other anthropogenically-complex landscapes and urban wildlife systems, the inclusion of urban landscape information facilitated two unexpected results. First, macaque movement was most accurately predicted in the central core of the island where the landscape is highly heterogeneous and mostly characterized by human landscape features. Second, urban landscapes mitigated pathogen transmission by reducing the infection rate of both pathogens in the more heterogeneous, urban landscape. This accuracy in modeling macaque dispersal provided the foundation for then comparing how the transmission of two closely related pathogens, but with two distinctly different severities, would be impacted by the landscape more broadly. We found that the inclusion of landscape data increased the distance from the initial site at which infection peaked in both parasites, but that in all cases, *E. dispar* successfully reached populations at further distances at higher rates. Due primarily to macaques favoring forest patches, infections outside of temple complexes occurred at the greatest rate in this landscape layer. Interestingly, *E. histolytica* had a significantly higher rate of transmission between macaques outside of temples. Thus, specific landscape features (forest patches and rice agriculture) allowed infection to thrive more than others (urban areas and buffer zones), favoring the more virulent pathogen even when the less virulent pathogen was able to more successfully reach other temple populations. This dynamic favors the spread of the less virulent pathogen to macaque populations, making it the more probable source of infections resulting from human-macaque interactions at temple sites, while the occasional, yet significant, human-macaque interactions occurring outside of temple sites is more likely to favor the transmission of the more virulent pathogen.

While LiNK focuses on the spatial patterns of host movement and gastrointestinal pathogen transmission patterns in an island population of long-tailed macaques, the results presented here demonstrate the importance of incorporating environmental components and landscape features more generally into models of pathogen transmission. Landscape heterogeneity is significant in shaping the pattern of both macaque dispersal and gut pathogen transmission across the island. We have shown that distance alone is not necessarily a significant indicator of transmission success, as demonstrated by the peaks in infection at further distances from individual populations. Rather, it is the combination of distance from initial infection site and landscape complexity that serve as the best predictor of infection patterns. We also demonstrate that some level of landscape heterogeneity serves to slow the spatial dimension of infection, which has important implications for land use management. Within the context of bidirectional pathogen transmission between humans and non-human primates, the significance of landscape dynamics on pathogen transmission potential could be even more significant when considering the rare, but important, occurrence of novel infectious disease emergence. Increasing human-wildlife interactions make it important to consider the impact of landscape complexity on pathogen transmission patterns, which can facilitate infectious disease emergences.

Establishing how patterns of infection vary spatially represents a key first step in understanding the ecological and epidemiological links between anthropogenic land use and disease [15]. ABMs are one tool for understanding how varying land uses can impact the spatial pattern of infection. Given this, the potential for ABMs to be used as a tool in the development of a disease management strategy, either a chronic invasive pathogen such as bovine tuberculosis in South Africa [57] or in response to a more acute outbreak such as influenza [58], is significant. Disease management strategies include large-scale vaccination efforts, administration of therapeutics, or often culling of infected individuals [59,60]. However, these efforts can be challenging. Vaccination efforts and administration of therapeutics often end when funding issues arise [61]. Culling has been shown to be often inefficient and ineffective [62-65]. Moreover, these efforts often have unintended ecological and evolutionary consequences [62-65]. However, the incorporation of complex landscape information and host behaviors into the planning of disease management strategies can provide a more realistic representation of the spatial pattern of infection.

Understanding the role of complexity is one of the most significant aspects in the effective analysis of infectious disease dynamics [66]. Recently, we have demonstrated that the landscape, including anthropogenic elements of the landscape, has an important factor in explaining infection intensity of specific parasites [35]. By identifying the emergent properties of populations and landscapes based on the decisions of individual hosts, LiNK provides a basis for understanding how the complexities of the anthropogenic landscape influence patterns of pathogen transmission at the scale of individuals, populations, and metapopulations. LiNK’s success at modeling actual dispersal distances in a complex landscape demonstrates the utility of ABMs in predicting patterns of host dispersal and pathogen transmission. Further, while the focus of this analysis was on the spatial patterns of transmission of gastrointestinal pathogens, which have direct life cycles, ABMs incorporating complex landscapes have also been utilized for both directly and indirectly infecting pathogens, successfully incorporating intermediate hosts as well [10,67]. For example, the spatial spread of Human African Trypanosomiasis was successfully modeled using an ABM, which incorporated human behavior and both the density and movement of the disease vector – the tsetse fly (*Glossina* spp.) [10]. Thus, ABMs can be informative for disease management by enabling researchers to model the behavioral patterns of hosts, reservoirs, and vectors, across increasingly complex landscapes.

## Conclusion

In summary, GIS-enabled agent-based models, such as LiNK, are capable of including important information regarding landscape heterogeneity and host behavior and are thus able to make accurate predictions about host dispersal and pathogen transmission patterns in complex landscapes. The significance of incorporating both host movement and landscape information into predictive models has previously been unrecognized. For example, without the inclusion of this data into our model and analyses, how the landscape and host movement together favor a less virulent pathogen within macaque populations, while concurrently allowing for the occasional, yet significant transmission of the more virulent pathogen in dispersing macaques would not have emerged as a relevant, and significant, finding. Utilizing these layers of additional data in modeling has the potential to target disease management efforts, identify areas of cryptic transmission events, and foresee potential unintended ecological consequences of disease management strategy implementation, especially in complex, anthropogenic landscapes. While our analysis focused on directly transmitted parasites, ABMs have been successfully implemented for parasites with more complex, indirect life cycles. Thus, GIS-enabled ABMs have the potential to inform management decisions and policies developed in response to both disease outbreaks and chronic, invasive pathogens in wildlife populations.

## Abbreviations

LiNK: Named for the authors of the model Lane, Neiderweiser, and Kennedy, this is the agent-based model developed to understand spatial and temporal pathogen transmission patterns; GIS: Geographic Information Systems; ABM: Agent-based Model; ODD: Overview Design concepts, and Details.

## Competing interests

The authors declare that they have no competing interests.

## Authors’ contributions

KELD was involved in all aspects of this manuscript, including funding, experimental design, model development and data analysis, post processing development, discussion and writing. RCK was involved in experimental design, model development and data analysis, post processing development, discussion and writing. SMNA was involved in experimental design, model development, post processing development and thoughtful discussion. GRM was involved in experimental design, model development, and thoughtful discussion. HH was involved in funding, experimental design, model development and data analysis, discussion and writing. AF was involved in funding, experimental design, model development and data analysis, discussion and writing. All authors read and approved the final manuscript.

## Supplementary Material

Additional file 1 **ODD Protocol **[28,30,31,33,44,52,68]**.** Click here for file

Additional file 2 Biological Relevance Verification & Validation Results. Click here for file
