# Supplementary material for: A test of agent-based models as a tool for predicting patterns of pathogen transmission in complex landscapes
Source: BMC Ecol. 2013 Sep 25;13:35. doi: 10.1186/1472-6785-13-35 (PMC3850893; doi:10.1186/1472-6785-13-35)
Supplement: Additional file 1 — ODD Protocol [28,30,31,33,44,52,68]. [file 1472-6785-13-35-S1.docx]

Appendix A: **ODD Protocol**

1. **Purpose**

The purpose of the LiNK simulation model is to help increase understanding of the effect of landscape on the spread of pathogens among long-tailed macaques on Bali, Indonesia.

1. **Entities, state variables and scales**

Entities within LiNK include agents representing macaques and the landscape. Our spatially explicit model includes the following landscape data (layers): coastline, cities, forests, lakes, rice fields, rivers, roads, and temple sites. We also include 3 buffer layers to represent the impact of humans and water on pathogenicity.

Macaque agents have a number of state variables, including sex, age, location, and whether infected. A full list of entities, their state variables, and model parameters in LiNK is included in Table A1. Notably, adult male macaque agents can disperse throughout the island, while female macaques spend their entire life within temples.

GIS data is utilized by the male macaques to make informed dispersal decisions. Specifically, we utilize ESRI shapefiles to represent landscape data. Because of performance considerations described in Kennedy [28], the shapefiles were used to create a grid-based system on which the macaques move. We utilized spatial queries of the landscape data to generate grids with a cell size of 111 meters. Each grid cell can consist of multiple types of landscape, where appropriate. Macaques also move within a given grid cell and possess a random directional bias. At each time step, which we model as 12 hours, a macaque surveys potential new positions, noting its current landscape and current directional bias. Each potential new location is assigned a value, which is later normalized. Movement is probability-based and relies (by default) on a Moore neighborhood. The range of values in Table A2 represents a weighted probability that a macaque will move from one landscape to another, with probabilities adapted from published macaque behavior [30, 31]. A simplified view of movement from a macaque’s perspective is show in Figure A1.

1. **Process overview and scheduling**

The LiNK model is event-driven; at each time step, a specified number of events are scheduled and executed. At the end of this section, we have included pseudocode for the main algorithms to clarify the scheduling we next describe. The pseudocode corresponds to the actual code used and the model could be re-implemented from this code. State variables from Table A1 are utilized in the pseudocode. A high level schematic for the life cycle of a macaque, as simulated through the schedule, is shown in Figure A2. High-level descriptions of the processes at each time step are described in the following paragraphs. We next provide a list of the processes and their order.

1. Dispersing macaques
   1. Increment age
      1. Potential to die from old age or infection thresholds
   2. Movement
      1. Potential to enter a temple if nearby
   3. Simulate Pathogen
      1. Potential to transmit pathogen
      2. Potential to die from pathogen
2. Macaques within temples
   1. Increment age
      1. Potential to die from old age or infection thresholds
   2. Can leave current temple if previous movement resulted in coordinates outside of temple
   3. Female macaques have the potential to give birth
   4. Simulate pathogen
      1. Potential to transmit pathogen
      2. Potential to die from pathogen
   5. Movement

Behavior for individual macaques depends on a number of factors, including age and sex. It is simplest to consider two groups of macaques: dispersing macaques and macaques within temples. Dispersing macaques are considered before macaques within temples. First, a macaque’s age is incremented, with the possibility for death if age or infection thresholds are met. Second, the macaque can move according to the probabilities described earlier. If a macaque is near enough to a temple (1 grid cell, or 111 meters by default), it can enter the temple. Finally, a macaque can transmit or die from infection, according to user-defined parameters. At this point, death can also occur as a result of dispersal or age. Within temples, macaques follow a similar schedule. Again, first, a macaque’s age is incremented, with possibility for death if age or infection thresholds are met. Male macaques have a maximum lifespan of 18 years and female macaques have a maximum lifespan of 20 years. Second, if the macaque’s previous coordinates exceed those of the temple boundaries and if the macaque is a male of dispersing age (5-8 years), then the macaque can leave the temple according to a probabilistic algorithm described in the pseudocode. Simply, a male macaque has the opportunity to leave a temple once per month; for each of these opportunities, the macaque has a roughly 18% chance to leave. Third, female macaques have a 25% chance to give birth annually from 3-13 years of age; this is normalized and considered at each time step. Fourth, infection is simulated, again according to user-defined parameters. Finally, macaques randomly move within the temples.

**Algorithm Schedule**

//The algorithm represents the overall scheduling for the model

Let *L* be the set of layers

Let *templeSites* be the set of temple site ids, each with an initial number of macaques

Let *numberOfInitialDispersedMacaques* be the initial number of dispersed macaques

Let **newMacaque()** be a function that creates an empty macaque agent

Let *b_1_* be the directional bias

Let **RandomInt**(*x*,*y*) return an integer from a uniform distribution of integers between *x* and *y*, inclusive

Let **setLocation**(*x*,*y*) set a macaque’s *x*- and *y*-coordinates

Let **RandomLatitude**() return a valid latitude within the bounds of Bali

Let **RandomLongitude**() return a valid longitude within the bounds of Bali

Let *numberOfTemples* be the number of temple sites

Let **newTemple()** be a function that creates an empty temple agent

Let *infectedTemple* be the id of the initially infected temple

Let *t* be the current timestep (corresponds to 12 hours)

Let *T* be the total number of timesteps

model.**load**(*L*)

model.**load**(*templeSites*)

//create dispersed macaques, set their directional bias, set their location,

// and set their age to be somewhere between 8 and 18 years old

**for** i=0 to *numberOfInitialDispersedMacaques* **do**

m 🡨 **newMacaque**()

m.*b_1_* 🡨 **RandomInt**(0,3)

//set age to randomly be between 8 and 18 years

m.age 🡨 **RandomInt**(365 * 2 * 8, 365 * 2 * 18)

m.**setLocation**(**RandomLatitude**(),**RandomLongitude**())

**end for**

//create each temple and populate with the appropriate number of macaques,

// setting a percentage as infected if the temple is configured to start with

// infection

**for** j = 0 to *numberOfTemples* **do**

temple 🡨 **newTemple**()

temple.numberOfMacaques 🡨 templeSites.getId(*j*).numberOfMacaques

**for** k = 0 to *temple.numberOfMacaques* **do**

m 🡨 **newMacaque**()

m.natalTemple 🡨 j

**if** j == *infectedTemple* **then**

**if RandomInt**(0,100) < 40 **then**

m.infected 🡨 true

**end if**

**end if**

templeMacaques.**add**(m)

**end for**

Temples.**add**(temple)

**end for**

//for each time step, simulate movement and the pathogen for all macaques

*t* 🡨 0

**while** *t* < *T* **do**

**dispersedMove()**

**for each** *temple* ∈ *Temples* **do**

temple.**templeMove()**

**end for**

*t* 🡨 *t* + 1

**end while**

**Algorithm dispersedMove**

//This algorithm contains the logic for dispersed macaque movement

Let *M* be the set of dispersed macaques

Let **RandomInt**(*x*,*y*) return an integer from a uniform distribution of integers between *x* and *y*, inclusive

Let *L_t+1_* be the set of possible locations for the next timestep

Let *l_t+1_* be the new location

Let *b_1_* be the directional bias

Let *b_2_* be the landscape bias

Let **Select**() be a function that selects the new location based on the normalized weights for each location *l*

Let **RandomDouble**(*x*,*y*) return an double from a uniform distribution of doubles between *x* and *y*, inclusive

Let **nearbyTemples**(*m*) return a set of temples near macaque *m*

Let **distance**(*x*,*y*) return the distance between agent *x* and agent *y*

Let *templeThreshold* be the threshold that a macaque must be within to move into a temple (defaults to 1 grid cell, which correlates to 111 meters)

//for every dispersed macaque

**for** **each** *m* ∈ *M* **do**

*m*.age 🡨 *m*.age + 1

**if** *m*.immune **then**

**if** *m*.acquiredImmunity > acquiredImmunity **then**

*m*.immune 🡨 false

**end if**

*m*.acquiredImmunity 🡨 *m*.acquiredImmunity + 1

**end if**

//a higher virulence means a decreased chance to move

**if** virulence < 80 || **RandomInt**(0,100) > virulence **then**

**if** *m*.currentLandscape == bufferLayer **then**

*m*.infectivity 🡨 infectivity * *m*.currentLandscapeGradient

**end if**

**for** all *l* ∈ *L_t+1_* **do**

*l* ← *b_1_* + *b_2_*

**end** **for**

*m*.*l_t+1_* ← **Select**(*l* ∈ *L_t+1_*)

*m*.*b_1_*.update

*m*.*b_2_*.update

*m*.currentLandscape.update

**end if**

**if** *m*.infected == true **then**

**dispersedSimulatePathogen**(*m*)

**end if**

**if RandomDouble**(0,1) < dispersalDeathsPerDay **then**

*m*.die

**end if**

**if** *m*.infected == true **then**

//virulence has a small chance to kill a macaque every timestep

**if RandomDouble**(0,100000) < virulence **then**

*m*.die

**end if**

**end if**

**if** *m*.age > maxAge **then**

*m*.die

**end if**

**if RandomInt**(0,100) > virulence **then**

*m*.NearbyTemples 🡨 **nearbyTemples**(*m*)

//join a temple if it is close enough

**for each** temple ∈ *m*.NearbyTemples

**if distance**(*m*,temple) < *templeThreshold* **then**

**if** temple != *m*.natalTemple **then**

*m*.joinTemple(temple)

**end if**

**end if**

**end for**

**end if**

**end for**

**Algorithm dispersedSimulatePathogen(*m*)**

//This algorithm simulates the pathogen for dispersing macaques

Let *m* be the macaque that simulates the pathogen

Let *M* be the set of dispersed macaques

Let **distance**(*x*,*y*) return the distance between agent *x* and agent *y*

Let **RandomInt**(*x*,*y*) return an integer from a uniform distribution of integers between *x* and *y*, inclusive

*m*.sickSteps 🡨 + 1

//macaques become symptomatic if their number of sick timesteps is greater

// than the latency

**if** *m*.sickSteps > latency **then**

*m*.symptomatic == true

**end if**

//for a macaque *m2* in the set of dispersed macaques, if its distance is

// within the infectivity ring of macaque m and if the infectiousness is

// appropriate then mark *m2* as infected

**for** **each** *m2* ∈ *M* **do**

**if** !*m2*.immune **then**

**if distance**(*m*,*m2*) < m.infectivity * 0.001 **then**

**if RandomInt**(0,100) ≤ *infectiousness* **then**

*m2*.infected 🡨 true

*m2*.sickSteps 🡨 0

**end if**

**end if**

**end if**

//if the pathogen has cleared, update macaque pathogen variables

**if** m*.*sickSteps > clearanceTime **then**

*m*.infected 🡨 false

*m*.symptomatic 🡨 false

*m*.immune 🡨 true

*m*.acquiredImmunity 🡨 0

**end if**

**end for**

**Algorithm templeMove**

//This algorithm simulates movement within each temple site

Let *M* be the number of macaques at this temple

Let **RandomDouble**(*x*,*y*) return an double from a uniform distribution of doubles between *x* and *y*, inclusive

Let **withinTemple**(*m*) be a function that returns true if macaque *m* is within the temple bounds and false otherwise

Let **RandomInt**(*x*,*y*) return an integer from a uniform distribution of integers between *x* and *y*, inclusive

Let **giveBirth**() be a function that creates a new macaque at the current temple with properties inherited from its mother, including the natal temple as the current temple and with a gender of equal chance being male or female

Let **setLocation**(*x*,*y*) set a macaque’s *x*- and *y*-coordinates

Let *xSize* and *ySize* be the width and length of the temple

Let **getLocationX**() and **getLocationY**() return the *x*- and *y*-coordinates for a temple, respectively

Let *b_1_* be the directional bias

//for every macaque *m* at a given temple

**for** **each** *m* ∈ *M* **do**

*m*.age 🡨 *m*.age + 1

**if** *m*.age > maxAge **then**

*m*.die

**end if**

//virulence kills a small number of macaques each time step

**if** *m*.infected && **RandomDouble**(0,100000) < virulence) **then**

*m*.die

**end if**

//if a mature male macaque (between 6 and 9 years old) has attempted to

// move beyond the temple bounds, allow him to leave a small percentage of

// the time

**if** !**withinTemple**(*m*) **then**

**if** *m*.sex == male && *m*.mature == true **then**

//male macaques have the opportunity to leave about a temple once per

// month, and then can leave about 18% of the time

**if** *m*.age % 60 == 0 **then**

**if RandomInt**(0,11) < 2 **then**

*m*.remove 🡨 true

**end if**

**end if**

**end if**

**end if**

//if the macaque stays in the temple

**if** !*m*.remove **then**

//let a percentage of mature female macaques (between 3 and 13 years old)

// give birth

**if** *m*.sex == female && *m*.mature == true && *m*.age % (365 * 2) == 0 **then**

**if RandomInt**(0,3) == 3 **then**

*m*.**giveBirth**()

**end if**

**end if**

**if** *m*.immune **then**

**if** *m*.acquiredImmunity > acquiredImmunity **then**

*m*.immune 🡨 false

**end if**

*m*.acquiredImmunity 🡨 *m*.acquiredImmunity + 1

**end if**

**if** *m*.infected **then**

**templeSimulatePathogen**(*m*)

**if** *m*.sickSteps > clearanceTime **then**

*m*.infected 🡨 false

*m*.symptomatic 🡨 false

*m*.immune 🡨 true

*m*.acquiredImmunity 🡨 0

**end if**

**end if**

**if** virulence < 80 || **RandomInt**(0,100) > virulence **then**

*m*.**setLocation**(*m*.**getX**() + **RandomInt**(-1,1), *m*.**getY**() + **RandomInt**(-1,1))

**end if**

**if** *m*.sex == female and !**withinTemple**(*m*) **then**

*m*.**setLocation**(*xSize*,*ySize*)

**end if**

**else**

*m*.setLocation(temple.**getLocationX**(),temple.**getLocationY**())

*m*.*b_1_* 🡨 **RandomInt**(0,3)

**end if**

**end for**

**Algorithm templeSimulatePathogen(*m*)**

//This algorithm simulates the pathogen within a temple site for a macaque *m*

Let *m* be the macaque that simulates the pathogen

Let **getMooreNeighbors**(*m*) return the Moore neighbors for macaque *m*

Let **RandomInt**(*x*,*y*) return an integer from a uniform distribution of integers between *x* and *y*, inclusive

*m*.sickSteps 🡨 *m*.sickSteps + 1

**if** *m*.sickSteps > latency **then**

*m*.symptomatic 🡨 true

**end if**

neighborMacaques 🡨 **getMooreNeighbors**(*m*)

**for** **each** *neighborMacaque* ∈ *neighborMacaques* **do**

**if RandomInt**(0,100) ≤ infectiousness **then**

**if** !neighborMacaque.immune **then**

neighborMacaque.infected 🡨 true

neighborMacaque.sickSteps 🡨 0

**end if**

**end if**

**end for**

1. **Design concepts**

*Basic Principles:* Two main principles influence the model’s design. Macaque behavior in reaction to landscape (habitat preference) has been studied [31] and was incorporated into the model through weighted movement probabilities. The second principle was the mechanism by which we modeled a given pathogen. Each of these principles are utilized as submodels and described in detail later. Their inclusion was necessary to facilitate the overall purpose of the model.

*Emergence:* Patterns of infection spread throughout Bali emerge over time, in accordance to host movement through the given landscape and according pathogen parameters. Landscape can be considered as individual layers or combinations of several layers.

*Adaptation*: Macaques have several adaptive traits. Females are only able to give birth between 3 and 13 years of age. Male macaques can only disperse between the ages of 5 and 8. Pathogens also adapt and influence the host macaque (and other macaques) in a manner described temporally in Figure A3.

*Objectives*: Macaques have no overarching desire for fitness in the LiNK model; instead, dispersing macaques have habitat preferences.

*Learning*: Macaques do not learn in the LiNK model

*Prediction*: Macaques do not estimate the success of a movement decision; rather, the movement is based on a weighted probability for a given landscape.

*Sensing:* Macaques are aware of their environment, which includes landscape data and other macaques. Their surroundings are used to make informed movement decisions and influence pathogen spread.

*Interaction:* Macaques interact with other macaques both while dispersing and within temples. When a macaque is within the ring of infectiousness of an infected macaque, it has the possibility to become infected. Macaques also interact with their environment. While a macaque does not explicitly change landscape, landscape influences macaque dispersal.

*Stochasticity:* Survival in the model is stochastic; pathogens and dispersal directly affect survival rate. Movement is also probability based. Certain landscapes are more desirable than others, and macaques move with a directional bias. Both landscape and directional bias factor into movement decisions. Births are stochastic, with females having an annual 25% chance of giving birth between the ages of 3 and 13. The sex of the offspring is 50% male and 50% female. Macaques located within temples often attempt to move beyond the bounds of the temple, which is permissible only a small percentage of the time and only for males of a specified age.

*Collectives*: Macaques do not belong to aggregations. Landscape data can be aggregated; zero to all landscape layers can be enabled for a given simulation. Movement amongst the layers is determined according to a weighted probability, regardless of the number of layers enabled.

*Observation:* Data is collected based on events. Each infection, death, birth, and movement between a temple and dispersal for every macaque is recorded in the output file for every macaque. The model is observed through its GUI and through analysis of the output file. LiNKStat, a LiNK specific analysis program, was written to process and present output [33].

1. **Initialization**

Upon initialization, both the landscape layers and the number of macaques within each temple site are constant and are based on previously collected data [31, 44, 52]. However, a simulation can be started with any number of landscape layers enabled. The initial geographic placement of macaques, both inside the temples and dispersing, is random. Values of individual pathogen parameters are selected by the user at the outset of each run. We have included the initial pathogen parameters for the pathogens modeled in this paper in Table A1.

1. **Input data**

The input to the model includes the GIS shapefiles representing the various landscape features of Bali [68]. We also utilize real-world population data to populate each temple with the appropriate number of macaques [30].

1. **Submodels**

*Pathogens:* Pathogen parameters were chosen with the assistance of domain experts with considerable expertise in the field. LiNK pathogen parameters were included in the model because of their ability to identify and differentiate individual and directly transmitted pathogens, allowing for the specificity needed to model unique pathogens while maintaining the flexibility to model a variety of pathogens. Upon infection, a pathogen is in a latent period, which refers to the length of time before the infection becomes symptomatic. Individuals can infect other individuals during the symptomatic phase of infection, which is between the end of the latency period and beginning of the immunity period. After completing the symptomatic phase, a macaque will become infection free and clear of the pathogen, which prevents further transmission of the pathogen. Immunity development occurs after the infection has been cleared from individual macaques, ending when the macaque again becomes susceptible. Transmission of the pathogen between macaques depends largely on infectiousness and infectivity. Infectivity is described as the transmission ring which both macaques have to be within to transfer infection; infectiousness is the likelihood that an infection will occur. Virulence is a measure of the severity of infection. Figure A3 shows the temporal relationships for selected pathogen-related states. A transition diagram for selected pathogen parameters is shown in Figure A4. Pathogen simulation is described in the scheduling pseudocode.

*Movement:* Macaque movement through the landscape was implemented based on previous findings [31, 52], including dispersal distances and habitat preferences. Male dispersal and female philopatry was confirmed through sequencing of mt and Y DNA loci [31]. The higher the virulence of an infected macaque, the smaller the chance that macaque will move. While movement within temples is random, movement amongst dispersing macaques is complex. In its simplest form, macaques survey their surroundings and current landscape at each time step, while also considering their previous direction of movement. Next, using a weighted probability described earlier a macaque chooses it next location, which includes the possibility of not moving. The mechanism of movement is independent of the number of layers enabled for any given simulation run, but the probabilities rely on the enabled layers. Pseudocode for macaque dispersal in the model is can be found in section 3 of this ODD.

Figure Legends:

Figure A1. Macaque Movement. The figure above represents a high-level view of how a macaque *M* determines where to move in a landscape consisting of forests (green) and a river (blue). Movement probabilities are associated with landscape features; for example, a macaque is more likely to enter a forest than a river.

Figure A2. Life cycle Transition Diagram. Macaques are always born in temple sites. Female macaques spend their entire lives within their natal temple, but mature male macaques can disperse throughout the island, with the ability to join other, non-natal temples.

Figure A3. Temporal Relationship of Pathogen Parameters and Related Events. The diagram above shows the relationship between various pathogen parameters in our simulation. Depending on the parameters used, macaques can become permanently immune to the modeled pathogen.

Figure A4. Pathogen Transition Diagram. Macaques generally begin as susceptible and then transition to other states after being infected. Macaques with a symptomatic infection can become reinfected and macaques can reinfect themselves (autoinfection). An acquired immunity is gained after most infections, but may be lost after a specified amount of time.

Table A1:

State variable and parameter value, from ODD submodels, describing specific features of the model, on an entity-by-entity basis. State variables are provided in *italics*. Behavioral values of macaques taken from [31, 52].

| **State Value or Paramter, by Entity** | **Value** |
| --- | --- |
| **Model***:* |  |
| Dispersal Deaths per Day | Variable |
| Autoinfection | Variable |
| Initial Infected | Variable |
| Population Size | Conforms to real data |
| Timestep | 12 hours |
| Grid cell size | 111 meters |
| **Macaque***:* |  |
| Sex (adults) | 75% female, 25% male; Dispersers = 100% male |
| *Age* | 50% adult (8-18 yr male, 8-20 yr female); 50% juvenile (0-8 yrs) |
| *Latitude* | Random within island bounds |
| *Longitude* | Random within island bounds |
| *Natal Temple* | Random within island bounds, but in accordance with temple population sizes |
| *Directional bias* | Random |
| *Current Landscape* | Based on latitude and longitude |
| *Infected* | True, if infected |
| *Sick Steps* | Number of timesteps infected |
| *Symptomatic* | Whether symptomatic |
| **Pathogen***:* |  |
| Virulence | (0-100 range) |
| *Infectivity* | (0-100 range) |
| *Infectiousness* | (0-100 range) |
| *Clearance time* | Variable, in timesteps |
| *Latency* | Variable, in timesteps |
| *Natural resistance* | (0-100 range) |
| *Acquired immunity* | Variable, in timesteps |

Table A2:

The range of values in Table A2 represents a weighted probability that a macaque will move from one landscape to another, with probabilities adapted from published macaque behavior [31, 52]. Row headers represent the current landscape and column headers represent the potential new landscape.

|  | **City** | **Coast** | **Forest** | **Lake** | **Rice Field** | **River** | **Road** |
| --- | --- | --- | --- | --- | --- | --- | --- |
| **City** | 10-30 | 15-20 | 40-70 | 0 | 10-30 | 15-45 | 15-20 |
| **Coast** | 5-20 | 20-30 | 40-70 | 0 | 10-30 | 15-45 | 5-20 |
| **Forest** | 5-20 | 15-20 | 45-80 | 0 | 10-30 | 15-45 | 0-20 |
| **Rice Field** | 5-20 | 15-20 | 40-70 | 0 | 15-40 | 15-45 | 0-20 |
| **River** | 5-20 | 15-20 | 40-70 | 0 | 10-30 | 20-55 | 0-20 |
| **Road** | 5-20 | 15-20 | 40-70 | 0 | 10-30 | 15-45 | 5-30 |
