# Supplementary material for: A test of agent-based models as a tool for predicting patterns of pathogen transmission in complex landscapes
Source: BMC Ecol. 2013 Sep 25;13:35. doi: 10.1186/1472-6785-13-35 (PMC3850893; doi:10.1186/1472-6785-13-35)
Supplement: Additional file 2 — Biological Relevance Verification & Validation Results. [file 1472-6785-13-35-S2.docx]

Additional file 2: **Biological Relevance Verification & Validation Results**

*Baseline Landscape Inclusion Tests:*

*Overall Infection:* Infection was significantly greater from each initial infection site when landscape data was included (Figure B1, Table B2), demonstrating that landscape plays a significant role in infection dynamics. Population size and landscape heterogeneity were shown to be significant factors in determining infection, with large populations (AK and PU) having a significantly greater number of infections than small populations (MK and AN), and with significant differences in infection dynamics between heterogeneous and homogeneous populations, with both coast only and all layers included (Figure B1, Table B2).

*Sex:* Across all simulation analyses comparing male and female rates of infection, with and without the incorporation of landscape features (Figure B2, Table B3), only one relationship showed no significant difference. Specifically, the number of infections between males and females, from the initial infection site AN, with all GIS layers included. This is likely a direct effect of landscape inclusion combined with the effect of overall small population size.

*Death Proportions:* Comparisons of the effects of landscape data on death rates – due to age, dispersal, or infection – demonstrated significant differences in death rates in all categories (Figure B3, Table B4). Deaths due to dispersal and age were significantly greater from each initial infection site with no landscape data included, while the landscape inclusion resulted in a significantly greater number of deaths due to infection. When comparing the number of deaths by site of initial infection (Figure B4, Table B5), significant differences were shown at each site, with parasite deaths significantly less than dispersal and age deaths.

*Pathogen Parameterization Tests:*

*Overall Infection:* In the virulence analysis, high virulence was shown to result in a significantly lower infection rate than for other virulence levels (Figure B5, Table B6), supporting our hypothesis of reduced infection at high virulence levels due to reduced overall macaque movement. In the analysis of infectivity, significant differences in infection occurred in all scenarios except at MK, with the lowest levels of infectivity resulting in either the highest or lowest number infections. Interestingly, this all supports our hypothesis due to susceptibility of this factor to landscape heterogeneity. In the infectiousness analysis, the lowest level of infectiousness was shown to result in a significantly lower number of infections. Again, this supports our hypothesis by demonstrating an infection limitation to extremely close contact only.

*Sex:* Across all analyses, significant differences in rates of infection were found, with males infected at a greater rate in all scenarios (Figure B6, Table B7). When comparing the effects of virulence differences, lower levels of virulence resulted in a significantly greater number of infections. Higher infectivity resulted in significantly higher rates of infection in large populations while lower infectivity resulted in a significantly higher rate of infection in small populations. And higher infectiousness resulted in significantly higher numbers of infections. In all scenarios, these results support our hypotheses, lending validity to the model function.

**Figure Legends:**

Figure B1:

Sensitivity analysis comparing the number of infections occurring with and without the inclusion of GIS data, resulting from infection at 4 sites. Both t and p values are reported in Table B2 for 1) overall rate of infection by temple, 2) rate of infection between sites in heterogeneous and homogeneous populations, and 3) rate of infection between large and small population sizes.

Figure B2:

Sensitivity analysis comparing the number of infections occurring with and without the inclusion of GIS data, resulting from infection at 4 sites and partitioned by sex of host. Both t and p values are reported in Table B3 for 1) overall rate of infection by temple, 2) rate of infection between sites in heterogeneous and homogeneous populations, and 3) rate of infection between large and small population sizes.

Figure B3:

Sensitivity analysis that compares the number and type of deaths, occurring with and without the inclusion of landscape/GIS data, resulting from infection at 4 sites. Both t and p values are reported for comparing the result of including landscape data by type of death in Table B4 . ANOVA results comparing the number of overall deaths by population are reported in Table B5.

Figure B4:

Sensitivity analysis comparing the impact of individual GIS layers on infection rate, using ANOVA and Tukey’s HSD post hoc analysis). Each analysis is the comparison of the number of infections occurring in each layer of the GIS landscape, partitioned by initial site of infection (PU: F=73.991, p<2.2e^-16^, Forest ↑; AN: F=1.7035, p=0.1676, ns; AK: F=97.489, p<2.2e^-16^, Forest ↑; MK: F-10.265, p=2.622e^-6^, Forest ↑)

Figure B5:

Sensitivity analysis of the effects of a) virulence, b) infectivity, and c) infectiousness on the island-wide rate of infections, using ANOVA and Tukey’s HSD post hoc analysis. Analyses compare the number of infections by each level of pathogenicity parameter, partitioned by initial infection site. F and p values are reported in Table B6.


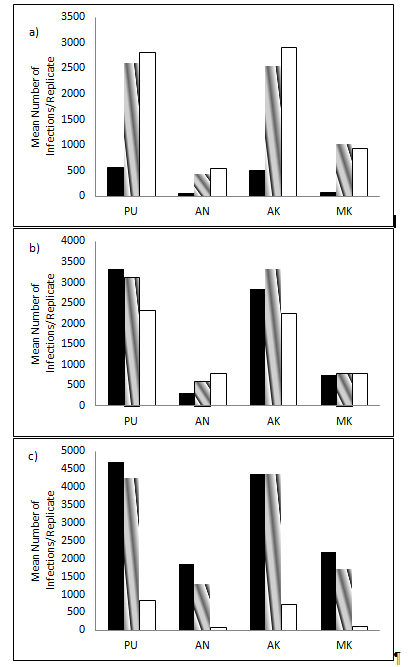


Figure B6:

Sensitivity analysis of the effects of a) virulence, b) infectivity, and c) infectiousness on the rate of infection in males and females. Analyses compare the number of infections in males vs females, originating from one of four initial infection sites. Table B7 reports t and p values, by strength of the pathogenicity parameter.

Table B2:

T-tests and p values associated with Figure B1: Effect of Landscape Inclusion on Infection. Degrees of freedom are 49 for landscape inclusion tests and 99 for both landscape heterogeneity and population size tests.

| **Comparison** | **t value** | **p value** |
| --- | --- | --- |
| ***Landscape Inclusion Tests*** |  |  |
| **PU** | 10.4757 | 2.499e^-11^ |
| **AN** | 2.7131 | 0.009171 |
| **AK** | 9.1166 | 3.955e^-12^ |
| **MK** | 4.0597 | 1.765e^-4^ |
| ***Landscape Heterogeneity Tests*** |  |  |
| **Heterogeneous: All GIS vs Coast Only** | 7.3581 | 5.533e^-11^ |
| **Homogeneous: All GIS vs Coast Only** | 8.336 | 4.552e^-13^ |
| **All GIS: Heterogeneous vs Homogeneous** | 7.5219 | 2.499e^-11^ |
| **Coast Only: Heterogeneous vs Homogeneous** | 8.806 | 4.373e^-14^ |
| ***Population Size Tests*** |  |  |
| **Large: All GIS vs Coast Only** | 16.368 | <2.2e^-16^ |
| **Small: All GIS vs Coast Only** | 4.7226 | 7.685e^-6^ |
| **All GIS: Large vs Small** | 4.2443 | 4.948e^-5^ |
| **Coast Only: Large vs Small** | 25.2564 | <2.2e^-16^ |

Table B3: T-tests and p values associated with Figure B2: Effect of Landscape Inclusion on Infections by Sex. Degrees of freedom are 49 for each analysis.

| **Comparison** | **t value** | **p value** |
| --- | --- | --- |
| ***PU:*** |  |  |
| **All GIS Layers:**  **Male vs Female** | 10.3704 | 5.936e^-14^ |
| **No GIS (only Coast):**  **Male vs Female** | 7.344 | 1.945e^-9^ |
| **Male_All GIS_ vs Male_No GIS_** | 8.9444 | 7.136e^-12^ |
| **Female_All GIS_vs Female_No GIS_** | 11.8357 | 5.605e^-16^ |
| ***AN:*** |  |  |
| **All GIS Layers:**  **Male vs Female** | 1.4473 | 0.1542 |
| **No GIS (only Coast):**  **Male vs Female** | 34.8328 | <2.2e^-16^ |
| **Male_All GIS_ vs Male_No GIS_** | 2.5055 | 0.0156 |
| **Female_All GIS_vs Female_No GIS_** | 2.9643 | 0.00674 |
| ***AK:*** |  |  |
| **All GIS Layers:**  **Male vs Female** | 11.4157 | 2.07e^-15^ |
| **No GIS (only Coast):**  **Male vs Female** | 9.0343 | 5.241e^-12^ |
| **Male_All GIS_ vs Male_No GIS_** | 11.8789 | 4.906e^-16^ |
| **Female_All GIS_vs Female_No GIS_** | 13.771 | <2.2e^-16^ |
| ***MK:*** |  |  |
| **All GIS Layers:**  **Male vs Female** | 3.1105 | 0.00311 |
| **No GIS (only Coast):**  **Male vs Female** | 15.6758 | <2.2e^-16^ |
| **Male_All GIS_ vs Male_No GIS_** | 3.9907 | 2.199e^-4^ |
| **Female_All GIS_vs Female_No GIS_** | 4.1846 | 1.181e^-4^ |

Table B4: T-tests and p values associated with Figure B3: Landscape Effects on Death Type Proportions. The effect the inclusion of landscape layers on each death type (age, dispersal risk, or infection). Each analysis is the comparison of the number of deaths with only the coast GIS layer included vs all GIS layers included in the analyses. Degrees of freedom are 49 for each analysis.

| **Comparison** | **t value** | **p value** |
| --- | --- | --- |
| ***PU:*** |  |  |
| **Age** | 150.603 | <2.2e^-16^ |
| **Dispersal** | 224.3567 | <2.2e^-16^ |
| **Parasite** | 11.9532 | 3.903e^-16^ |
| ***AN:*** |  |  |
| **Age** | 168.2958 | <2.2e^-16^ |
| **Dispersal** | 296.6979 | <2.2e^-16^ |
| **Parasite** | 2.8918 | 0.005697 |
| ***AK:*** |  |  |
| **Age** | 148.4287 | <2.2e^-16^ |
| **Dispersal** | 215.9748 | <2.2e^-16^ |
| **Parasite** | 13.2862 | <2.2e^-16^ |
| ***MK:*** |  |  |
| **Age** | 157.39 | <2.2e^-16^ |
| **Dispersal** | 233.1591 | <2.2e^-16^ |
| **Parasite** | 4.1269 | 1.422e^-4^ |

Table B5: Results of ANOVA comparing individual death types, as associated with Figure B3: Landscape Effects on Death Type Proportions. Each analysis is the comparison of the number of deaths by site of origination of infection.

| **Comparison** | **F value** | **p value** | **** Tukey HSD & Direction** |
| --- | --- | --- | --- |
| ***PU:*** |  |  |  |
| **All GIS** | 145.97 | <2.2e^-16^ | Parasite Deaths ↓ |
| **Coast Only** | 17914 | <2.2e^-16^ | Parasite Deaths ↓ |
| ***AN:*** |  |  |  |
| **All GIS** | 2304.7 | <2.2e^-16^ | Parasite Deaths ↓ |
| **Coast Only** | 52391 | <2.2e^-16^ | Parasite Deaths ↓ |
| ***AK:*** |  |  |  |
| **All GIS** | 154.18 | <2.2e^-16^ | Parasite Deaths ↓ |
| **Coast Only** | 18436 | <2.2e^-16^ | Parasite Deaths ↓ |
| ***MK:*** |  |  |  |
| **All GIS** | 625.99 | <2.2e^-16^ | Parasite Deaths ↓ |
| **Coast Only** | 51797 | <2.2e^-16^ | Parasite Deaths ↓ |

Table B6: *Pathogen parameterization test verification & validation:*

Results of ANOVA, comparing infection as a function of sensitivity to virulence, infectivity, and infectiousness parameters, associated with Figure B5: Effect of Virulence, Infectivity, and Infectiousness on Rate of Infection.

| **Comparison** | **F value** | **p value** | **** Tukey HSD & Direction** |
| --- | --- | --- | --- |
| ***Virulence:*** |  |  |  |
| **PU** | 52.704 | <2.2e^-16^ | High ↓ |
| **AN** | 4.4299 | 0.01355 | High ↓ |
| **AK** | 56.005 | <2.2e^-16^ | High ↓ |
| **MK** | 9.3479 | 1.508e^-4^ | High ↓ |
| ***Infectivity:*** |  |  |  |
| **PU** | 6.2056 | 2.586e^-3^ | Low ↓ |
| **AN** | 4.1585 | 0.01751 | Low ↑ |
| **AK** | 5.8499 | 3.593e^-3^ | Low ↓ |
| **MK** | 0.0112 | 0.9988 | n/s |
| ***Infectiousness:*** |  |  |  |
| **PU** | 303.76 | <2.2e^-16^ | Low ↓ |
| **AN** | 4.1597 | 0.01749 | Low ↓ |
| **AK** | 19.848 | 2.342e^-8^ | Low ↓ |
| **MK** | 42.341 | 3.008e^-15^ | Low ↓ |

Table B7: *Pathogen parameterization test verification & validation:*

T-tests and p values associated with Figure B6. Tests compare the effects of high, moderate, and low **virulence, infectivity, or infectiousness** levels on the number of individual males and females becoming infected, partitioned by the initial site of infection.

| **Analysis** |  | **t value** | **p value** |
| --- | --- | --- | --- |
| Virulence | PU |  |  |
|  | High | 2.855 | 0.006338 |
|  | Moderate | 9.3523 | 2.164e^-12^ |
|  | Low | 8.4647 | 4.410e^-11^ |
|  | AN |  |  |
|  | High | 57.8448 | <2.2e^-16^ |
|  | Moderate | 2.3396 | 0.02351 |
|  | Low | 2.4227 | 0.01923 |
|  | AK |  |  |
|  | High | 2.7166 | 0.009144 |
|  | Moderate | 8.6614 | 2.246e^-11^ |
|  | Low | 8.9099 | 9.626e^-12^ |
|  | MK |  |  |
|  | High | 69.2637 | <2.2e^-16^ |
|  | Moderate | 3.6356 | 6.75e^-4^ |
|  | Low | 3.515 | 9.707e^4^ |
| Infectivity | PU |  |  |
|  | High | 12.5219 | <2.2e^-16^ |
|  | Moderate | 9.5978 | 7.72e^-13^ |
|  | Low | 7.5035 | 1.105e^-9^ |
|  | AN |  |  |
|  | High | 2.0918 | 0.04166 |
|  | Moderate | 2.7107 | 0.00923 |
|  | Low | 3.0802 | 3.387e^-3^ |
|  | AK |  |  |
|  | High | 9.1694 | 3.302e^-12^ |
|  | Moderate | 11.7065 | 8.362e^-16^ |
|  | Low | 6.9307 | 8.493e^-9^ |
|  | MK |  |  |
|  | High | 2.8668 | 6.095e^-3^ |
|  | Moderate | 3.2328 | 2.196e^-3^ |
|  | Low | 2.7731 | 7.831e^-3^ |
| Infectiousness | PU |  |  |
|  | High | 22.0084 | <2.2e^-16^ |
|  | Moderate | 18.3199 | <2.2e^-16^ |
|  | Low | 5.5756 | 1.108e^-8^ |
|  | AN |  |  |
|  | High | 4.9721 | 8.864e^-6^ |
|  | Moderate | 4.2017 | 1.145e^-4^ |
|  | Low | 1.8675 | 0.06794 |
|  | AK |  |  |
|  | High | 17.2366 | <2.2e^-16^ |
|  | Moderate | 16.114 | <2.2e^-16^ |
|  | Low | 6.4123 | 5.862e^-8^ |
|  | MK |  |  |
|  | High | 6.1433 | 1.514e^-7^ |
|  | Moderate | 5.1467 | 4.882e^-6^ |
|  | Low | 2.4718 | 0.01704 |
